# Off‐iso Winston‐Lutz test on seven linear accelerators

**DOI:** 10.1002/acm2.14470

**Published:** 2024-07-23

**Authors:** Junfang Gao, David Anand

**Affiliations:** ^1^ Radiotherapy Clinics of Georgia Decatur Georgia USA; ^2^ Radiation oncology department, Texas Oncology Houston Texas USA

**Keywords:** multiple‐mets SRS, off‐iso Winston‐Lutz test, SIMT‐SRS, Winston‐Lutz‐Gao test

## Abstract

**Purpose:**

The aim of this study is to find optimal gantry, collimator, and couch angles for performing single isocenter, multiple target stereotactic radiosurgery (SIMT‐SRS). Nineteen angle sets were tested across seven linear accelerators for radiation‐isocenter coincidence and off‐isocenter coincidence. The off‐isocenter Winston‐Lutz test was performed to evaluate the accuracy of isocenter alignment for each angle set, and optimal angle sets as well as maximum off‐isocenter distance to target for each angle set was determined. The influence of simulated patient weight on off‐iso Winston‐Lutz test accuracy was also inspected.

**Method:**

The SNC MultiMet‐WL phantom and MultiMet‐WL QA Software v2.1 were used for the direct measurement and analysis of the off‐iso Winston‐Lutz test (also referred to as Winston‐Lutz‐Gao test). A two‐step method was developed to ensure precise initial placement of the target. Nineteen beams were delivered at 6X energy and 2 × 2 cm field size to each of six targets on the MultiMet Cube with couch kicks at five cardinal angles (90°, 45°, 0°, 315°, and 270°). To reduce imaging uncertainty, only EPID was used in target alignment and test image acquisition. A total of 200 Ibs (90.7 kg) of weight was also used to mimic patient weight. All tests were performed on both the free table and the weighted table.

**Results:**

For two new TrueBeam machines, the maximum offset was within the 1 mm tolerance when the off‐iso distance is less than 7 cm. Two older VitalBeam machines exhibited unfavorable gantry, couch, and collimator (GCC) angle sets: Linac No. 3 at (0,90,0), (0,270,0) and Linac No. 4 at (0,45,45) and (0,90,0). The C‐Series Linacs failed in the majority of GCC angle sets, with Linac No. 5 exhibiting a maximum offset of 1.53 mm. Four of seven machines show a clear trend that offset increases with off‐isocenter distance. Additionally, the IGRT table was less susceptible to the addition of simulated patient weight than the ExactCouch.

**Conclusion:**

Among the seven linear accelerators addressed, newer model machines such as the Varian TrueBeam were more precise than older models, especially in comparison to the C‐Series Linacs. The newer machines are more suitable for delivering SIMT‐SRS procedures in all GCC angle sets, and the results indicate that newer TrueBeams are capable of performing SIMT‐SRS procedures at all angle sets for targets of off‐iso distances up to 7 cm. The trend that offset between the target center and radiation field center increases with off‐iso distance, however, does not always hold true across machines. This may be comprised by the EPID's severe off‐axis horn effect. Lastly, the IGRT couch was less susceptible to patient weight compared to ExactCouch in the off‐isocenter Winston‐Lutz test.

## 1 INTRODUCTION

The off‐iso Winston‐Lutz test is designed to evaluate the accuracy of the off‐isocenter target coincidence accuracy between physical and radiation position in linear accelerator (Linac)‐based single isocenter multiple target stereotactic radiosurgery (SIMT‐SRS). Since it was first time proposed and performed by Gao and Liu^1^ in 2016, many medical physicists, researchers, and commercial companies have begun developing equipment and implementing this test in their clinic. Per naming convention, it is also referred to as Winston‐Lutz‐Gao (WLG) test. Some researchers or physicists unmethodically used the term “Off‐axis Winston‐Lutz test” to denote the content of the off‐iso Winston‐Lutz test. This is misleading and confusing because the Linac has three rotational axes—gantry, collimator, and couch—which point from—∞ to + ∞. Any target regardless of distance from the isocenter, which sits on three rotational axes will be ignored by the term “off‐axis”. The purpose of the off‐isocenter Winston‐Lutz test (WLG test) is to assess the positional accuracy of any target which is located away from the isocenter. This is a more clinically meaningful and precise definition. In this manuscript, the word “offset” is used to indicate the position difference between the target center and the radiation field center. The term, “off‐iso distance”, indicates the distance between the isocenter and off‐iso target center.

In 2016 Gao and Liu^1^ conducted first off‐iso Winston‐Lutz test on old Varian 21EX both collimated by jaw and MLC, respectively. It was shown that the offset increase with the off‐iso distance and concluded that the 3 cm is maximum off‐iso distance that could maintain coincidence within 1 mm offset. Gao and Liu emphasized that each clinic should conduct their own test to create planning guidelines for their respective machine. In same year of 2016, Wen et al.^2^ conducted a shared isocenter geometrical test for seven ceramic BBs with diameter of 5 mm under Varian Edge Linac with HD MLC. One BB was positioned at isocenter while other six BBs were positioned at various locations away from isocenter. Using classical image analysis, it was determined that offset increased with off‐iso distance in auto‐registration. There was no clear trend, however, for manual registration. In 2017 Gary Ezzell^3^ performed special positioning accuracy in ExacTrac and CBCT imaging system. One home‐made phantom has 12 BBs inside. This test is not off‐iso Winston‐Lutz test.

In 2018, Poder et al.^4^ modified the inside portion of the Perspex phantom from ArcCheck into a multi‐target single iso (MTSI) phantom to validate the position accuracy with CBCT alignment, and all BBs were irradiated through EPID from TrueBeam Millennium 120 MLC. The maximum off‐iso distance measured is 6 cm to achieve offset <1 mm accuracy. In the same year of 2018, Yaqoub^5^ conducted an automated Winston‐Lutz test at different off‐iso distance using a ball‐bearing (BB) of 5 mm diameter. The phantom can move at the precision of 0.01 mm by Vernier adjustment device. It was found that offset increases with off‐iso distance until 6 cm away from the isocenter, followed by a decrease at 8 cm. In 2019, Gao and Liu^6^ performed a similar test on TrueBeam STx treatment mode with BrainLab SRS target pointer with a 3.5 mm metallic ball built in the center. The target pointer can move at the precision of 0.1 mm. They found that 6 cm was the maximum distance from the isocenter that maintained the offset <1 mm. This was the first off‐isocenter Winston‐Lutz test to use simulated patient weight on the table.

In 2020, SunNuclear Corporation introduced the MultiMet‐WL QA phantom^7^ for the medical physics field to carry out off‐iso Winston‐lutz test exclusively. The phantom is made in 19.5 cm × 8.5 cm × 8.5 cm rigid, rectangular prism with six sphere BBs of 5.0 ± 0.025 mm diameter. The phantom can be either used independently or in conjunction with the StereoPhan device and comes with user‐friendly software for image analysis. In the same year of 2020, Capaldi et al.^8^ used a 3D printer to create a “3‐in‐1” integrated phantom for ion chamber dosimetry, film dosimetry, and off‐iso Winston‐lutz test. One 6 mm diameter BB is at the center and four 3 mm diameter BBs are located off‐center. They found a similar trend as Gao and Liu^6^ that the offset increases with off‐iso distance and the maximum off‐iso distance is 7 cm to maintain offset <1 mm.

In 2022, Pudsey et al.^9^ used SNC MultiMet‐WL phantom in their clinic and started off‐iso Winston‐lutz test. They inserted the cube phantom into StereoPhan which is mounted into QFix Encompass headboard. They investigated the rotational error from couch, gantry, collimator impact on the GTV and PTV's dosimetry coverage by intentionally introduced 0.5∼2.0‐degree error for three rotation axis. The analysis revealed that rotation error exceeding 0.5 degrees resulted in ∼1 mm offset when the off‐isocenter distance is larger than 5.8 cm (corresponding to Target 4 in this study). In the same year of 2022, Eagle et al.^10^ did off‐iso Winston‐Lutz test by the complicated three set coordinate transformation to obtain proper MLC shaping to center the BBs position. The method can compensate some deficit of using expensive VisionRT third party phantom, but the authors noted unknown initial position uncertainty for each target, even when measured from CT images. They concluded that the maximum offset between BB and radiation field was 0.72 mm. In the same year of 2022, Oliver et al.^11^ 3D printed one phantom which contains five tungsten carbide BBs with diameter of 2.38 mm. One special software was also built based on Hough circle detection algorithm. They did test on two different model Linac and did not see the clear trend that BBs’ offset increase with off‐iso distance.

In 2023, Li et al.^12^ used GafChromic films in a TrueBeam STX Linac for off‐isocenter Winston‐Lutz testing. By introducing intentional couch rotation errors of 0.5° and 1°, it was found that target offset increased with off‐isocenter distance up to 6 cm. The maximum offsets were 1.18 and 1.44 mm, respectively, for 0.5° and 1° couch rotation errors. Notably, when the distance reached 9 and 12 cm, the target offset decreased slightly and was more accurate, showing similar findings to those by Yaqoub.^5^ These findings will be further explored in the discussion section of this paper. In the same year of 2023, Rojas‐Lopez et al.^13^ used one anthropomorphic phantom to validate if both Etv6 and ETD two different ExacTrac system can correct the radiation and mechanical center offset through off‐iso Winston‐lutz test. Since the purpose and design of their study are different, it doesn't give much valuable data information on off‐iso Winston‐Lutz test itself.

Among all the studies above, none of them report the accuracy of initial placement, but it is known that the initial placement directly affects the final measurement results. Additionally, none of them discriminate between the pass or fail of gantry, couch, and collimator (GCC) angle sets in whole 3D space. In this study, the off‐isocenter Winston‐Lutz test was performed on seven linear accelerators to observe the relationship between the offset and off‐isocenter distance. The SNC MultiMet‐WL Cube phantom was placed directly on the table without integration with StereoPhan. A SunNuclear test treatment plan was modified for accuracy and used to deliver the off‐iso Winston‐Lutz test. The test was performed on seven Varian linear accelerators to evaluate the offset accuracy, with and without simulated patient weight on the table. A two‐step placement method was developed to ensure initial positioning accuracy on a submillimeter scale. Only the EPID system was utilized in the initial image alignment for phantom placement, minimizing the uncertainty from other imaging modalities (like kV, CBCT system, and Surface imaging).

## 2 MATERIAL AND METHODS

Seven linear accelerators are distributed across seven different clinical sites within a large metropolitan area. All seven Linacs offer single isocenter multiple target cranial SRS/SRT procedures for cancer patients and are equipped with kV, CBCT, and EPID image guidance systems. Linear accelerator performance depends not only on the model of the machine but also on the machine's age and workload. The year of commissioning and beam filament time are useful parameters for estimating these factors. The characteristics of all seven linear accelerators are summarized in Table 1.

**TABLE 1:** Model and clinical characteristics of seven linear accelerators.

Linac No.	1	2	3	4	5	6	7
Model	TrueBeam	TrueBeam	VitalBeam	VitalBeam	Trilogy	21iX	21iX
Year of Commis.	07.2023	08.2023	07.2018	08.2022	06.2008	05.2011	05.2012
Table Style	IGRT	IGRT	IGRT	IGRT	IGRT	IGRT	Exact
Fila. Time (hr)	2510	2600	18050	17116	50450	34740	41342
Surface imaging	VisionRT	VisionRT	N/A	N/A	N/A	N/A	N/A

**FIGURE 1:**
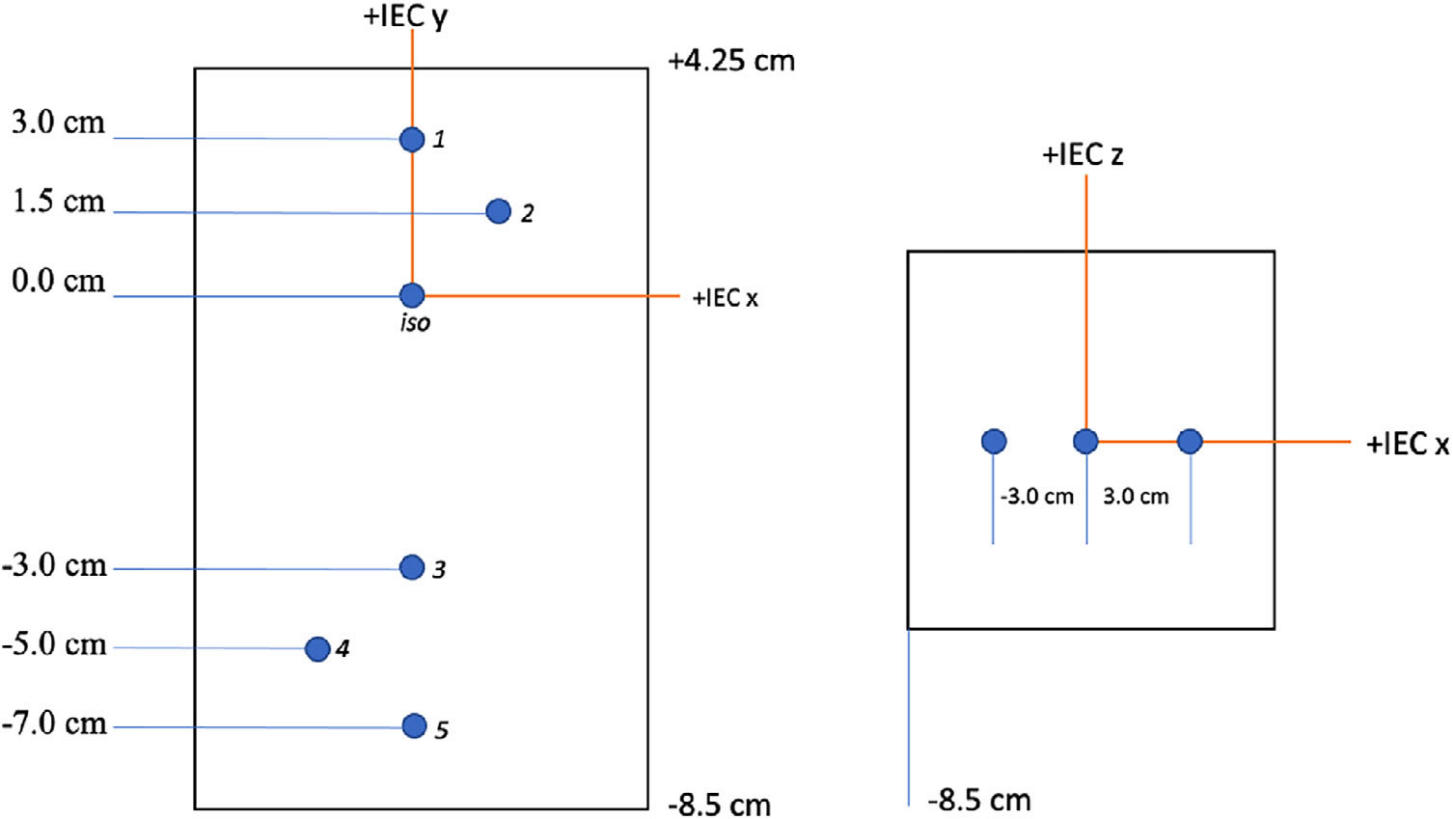
The AP and axial view sketch of SNC MultiMet‐WL Cube phantom.

SNC MultiMet‐WL system was chosen for the off‐iso Winston‐lutz test (WLG test) because of its design. The Multi‐Met WL Cube Phantom is built with six spherical tungsten carbide BBs placed within a uniform phantom (19.5 × 8.5 × 8.5 cm^3^) at measurement‐friendly distances with high precision (± 0.05 mm). The six BBs measure 5.0 ± 0.025 mm in diameter and are ideal for direct measurements of machine offset in the WLG test. The phantom eliminates the need for complicated mathematical coordinate transformations in treatment planning and delivery and reduces uncertainty from 3D printing phantoms. Its design is illustrated in Figure 1.

All test results were analyzed by the MultiMet‐WL QA Software v2.1, the associated software provided by Sun Nuclear Corporation. The software is designed for both Varian and Elekta devices and is compatible with two treatment plan options: a simplified test containing 10 fields and a more comprehensive test containing 19 fields. Depending on the treatment plan, the software can read either 10 or 19 DICOM images at once. A template treatment plan was obtained from the SNC Corporation website, and revisions were made to improve the target BB contours. Varian Linacs with Millennium 120 MLCs were configured at software setup and 45‐degree couch kicks were included in the comprehensive test option (19 DICOM images). The treatment plan was comprised of 19 dynamic step‐and‐shoot beams (As indicated in Table 2) designed to irradiate each target BB with a 2 cm × 2 cm square‐shaped field defined by the MLCs and delivering 50 MU per beam. During the processing, the IEC61217 standard coordinate system was selected for both 3D (X, Y, X) and 2D (VU) in order to maintain consistency with RV and Eclipse treatment planning systems. After image‐processing, a report was generated that included positional error data, results summary graphs, additional details. The beam configurations are as follows:

**TABLE 2:** Beam configuration of 19 beams.

Field	Gantry	Couch	Collimator	Target BBs
1	0	0	0	6
2	0	0	90	6
3	0	0	270	6
4	0	45	45	6
5	0	45	225	6
6	0	90	0	6
7	0	270	0	6
8	0	315	135	6
9	0	315	315	6
10	90	0	90	4
11	90	0	90	2
12	90	0	270	4
13	90	0	270	2
14	180	0	0	6
15	180	0	90	6
16	270	0	90	4
17	270	0	90	2
18	270	0	270	4
19	270	0	270	2

**FIGURE 2:**
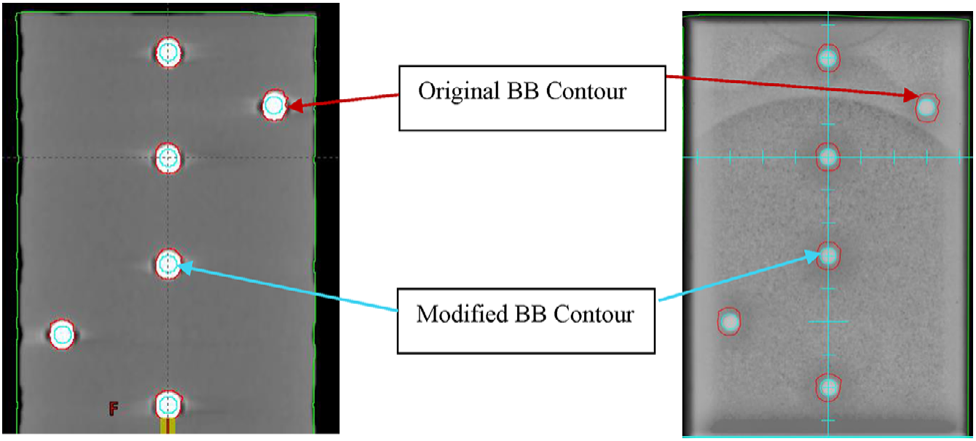
The original and modified BBs’ contour display on simulation CT (left) and portal image (right).

To minimize uncertainty from the imaging system, the EPID was used for both image‐based alignment of the phantom as well as data acquisition for the off‐isocenter Winston‐lutz test. This differs from SNC's recommendation to use CBCT or kV alignment. The original contours for the target BBs were inaccurate and contained severe artifacts due to tungsten carbide's high atomic number. Figure 2 shows a clear discrepancy between the contours and the BB's true size of 5 mm in diameter.

It is nearly impossible to accurately identify the center of each BB's location and expected 3D position using the original contours. Table 3 shows the planning position of each BB in the Eclipse TPS versus their expected position in the imaging space.

**TABLE 3:** Each target BBs planning position in Eclipse and expected position in image space.

BB No.	X (mm)	Y (mm)	Z (mm)	S (mm)
0 (iso)	0	0	0	0
1	0	30	0	30
2	30	15	0	33.54
3	0	−30	0	30
4	−30	−50	0	58.31
5	0	−70	0	70

Using the expected 3D position and the manufacturer's mechanical geometry specifications, new contours were created for each target BB. All BBs were then converted into high resolution contours in Eclipse. The center of each BB was placed on the physical position in Eclipse exactly, and the center of the No. 0 BB was chosen as the position of “planning isocenter.” This proved essential in achieving submillimeter precision during initial positioning. The accuracy of the initial placement of the phantom and targets is of crucial importance in the WLG test. A two‐step method was developed to position the target BBs. This method was based on a thorough understanding of the concepts of planning isocenter, imaging isocenter, mechanical isocenter, and radiation isocenter.

The *planning isocenter* is defined in the treatment planning system and is established after the user origin coordinate system is defined from the CT simulation. Planning isocenter is drawn in virtual space on the computer screen and rotational uncertainty is zero at any angle. The *image isocenter* is a physical point or small zone inside the treatment room that represents the central point around which the imaging system rotates. It is expected to be calibrated and represented by the image center on the 4DITC computer screen (for Varian Linac user) during initial commissioning and/or routine imaging QA. Each imaging system, including the MV portal imager, kV imager, and CBCT imager, has its own isocenter, but they should be coincident within an uncertainty tolerance through the IsoCal test (for Varian Linac user). The *mechanical isocenter* is defined as the physical cross point or small zone where the gantry rotational axis, collimator rotational axis, and couch rotational axis intersect. Accurately locating the mechanical isocenter is a challenging task.^14^ In clinical applications, two front pointers are often used to determine the mechanical isocenter. The *radiation isocenter* is the intersection point or zone where the central axis of radiation beams intersects during gantry and/or collimator rotation. It can be located by star shot using GafChromic film or EPID imager.

**FIGURE 3:**
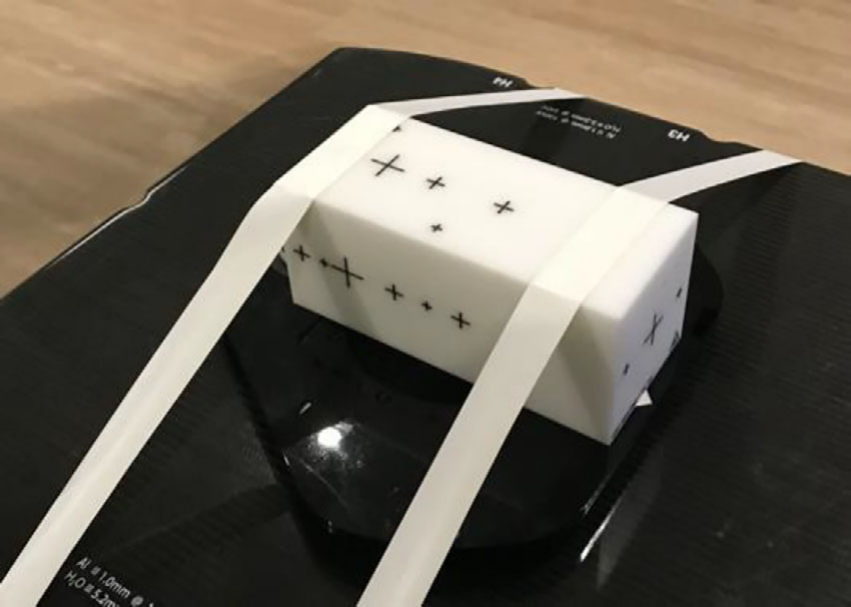
Demonstration of Phantom placement.

**FIGURE 4:**
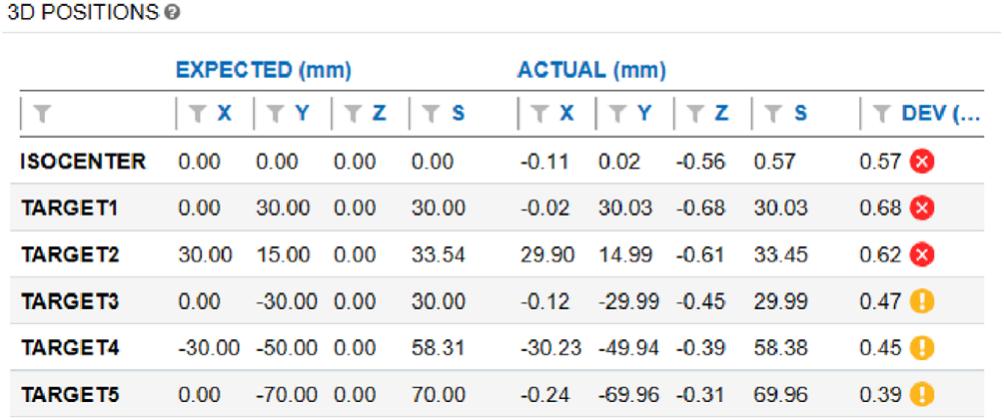
Demonstration of initial target placement results.

First step: The phantom was placed on the table and leveled in the cross‐plane and in‐plane directions. The isocenter target was then aligned very carefully with crosshairs as in Figure 3. AP and lateral portal images were acquired and manually aligned. At this point, the targets were taken to be aligned with the planning isocenter and its expected position because targets only match contours which are in the DRR image. The contours in the DRR image are from the Eclipse plan. Next, 19 images were acquired in accordance with the treatment plan and loaded into the MultiMet‐WL QA Software for analysis. The primary targets’ positional errors are displayed in Figure 4. The last column indicates the deviation from the expected position for each target (criteria: Pass < 0.3 mm, Acceptable < 0.5 mm). It is clear that the isocenter target, target 1, and target 2 were not positioned accurately.

**FIGURE 5:**
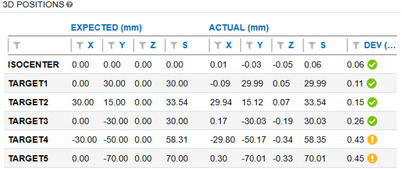
Demonstration of final target placement results.

Second step: the treatment table was returned to initial position and AP and lateral portal images were reacquired. The isocenter target was manually shifted to the (‐X, ‐Y, Z) position, and the treatment plan was redelivered as in the first step. The resulting position is displayed in Figure 5. After image reacquisition, all the targets showed positional errors within the acceptable criteria (<0.5 mm). Furthermore, the isocenter target is almost (0,0,0) which we believe is the image isocenter. This is much better positional accuracy than the 0.57 mm isocenter deviation shown in Figure 4. The challenge lies in accurately placing all the targets at their expected position. The isocenter BBs should be prioritized, and position error should be balanced among all targets.

One approximation: during the off‐isocenter Winston‐lutz test, it is assumed that the image isocenter, mechanical isocenter, and radiation isocenter have been coincident within an uncertainty through other acceptance test, and routine calibration procedures. Those tests are beyond the scope of this discussion.

**FIGURE 6:**
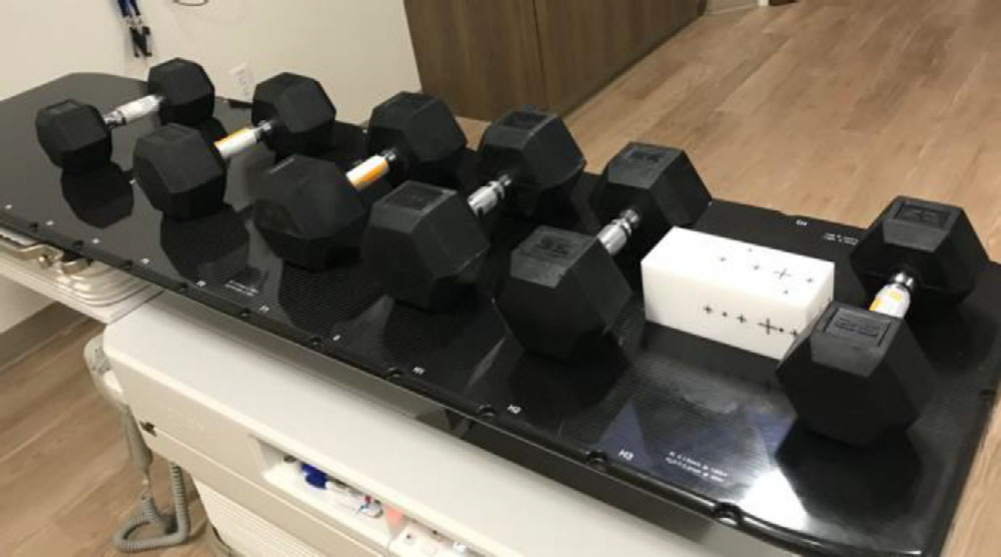
The 200 Ibs (90.7 kg) weight is distributed on the table as average patient weight.

This study also investigated the influence of patient weight on the accuracy of the off‐isocenter Winston‐Lutz test. For each machine, the test was performed on the free table and on a table with simulated patient weight (six dumbbells weighing a total of 200 pounds [90.7 kg]) were spread across the table in order to mimic real patients. They were distributed on the table as shown in Figure 6.

## 3 RESULTS

The test results of seven Linacs are presented in Figure 7. The machine number is listed on the left side. Left column are measurement results on the free table while the right column results are with 200 Ibs weight on it. The pass or fail of results are shown at the bottom of each picture.

FIGURE 7Off‐isocenter Winston‐Lutz test results on seven Liancs.

**Figure:**
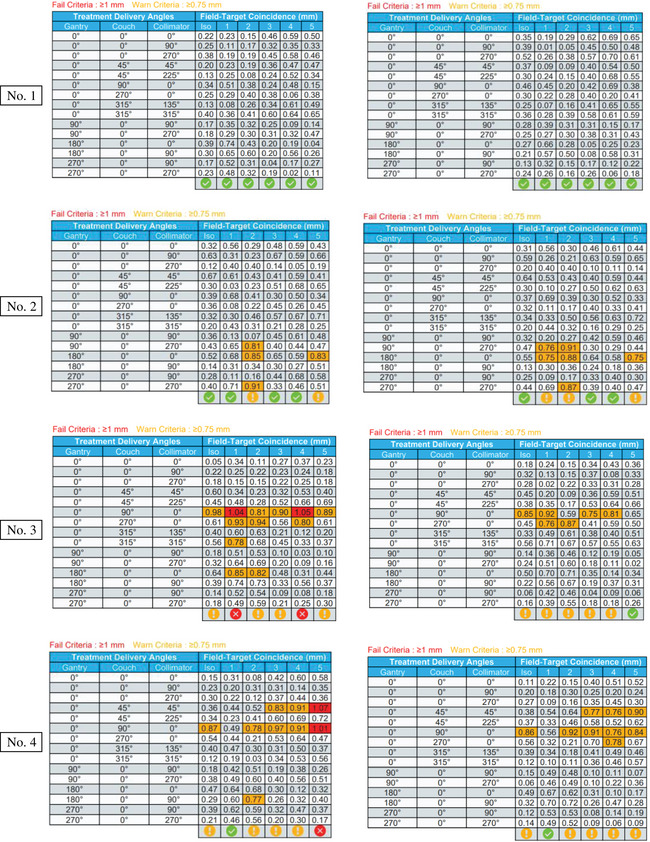

**Figure:**
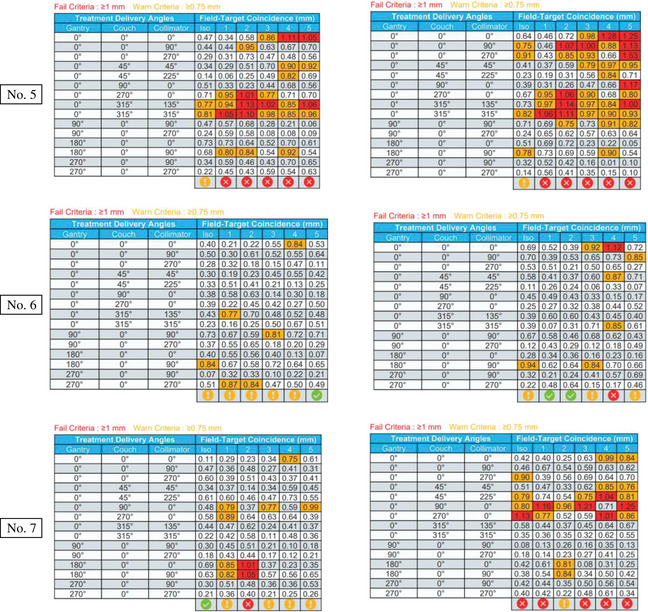

Figure 8 shows the average offset between target isocenter and radiation field center as a function of off‐isocenter distance. The measurement results for all GCC angle sets were averaged for each machine. Error bars representing one standard deviation were included in each plot. To investigate the relationship between offset and off‐isocenter distance, only the results for target isocenter (0 cm), target 3 (3 cm from iso) and target 5 (7 cm from iso) are shown. Data from targets 2 and 4 are not included in this plot due to roll involvement. The machine number was labeled inside the plot as No.* for free table and as No.*_W for weight on table.

FIGURE 8Offset vs. off‐isocenter distance plotted on the average gantry, couch, and collimator (GCC) angles for seven Linacs. No.* indicates free table and No.*_W indicates table with 200 pounds of weight.

**Figure:**
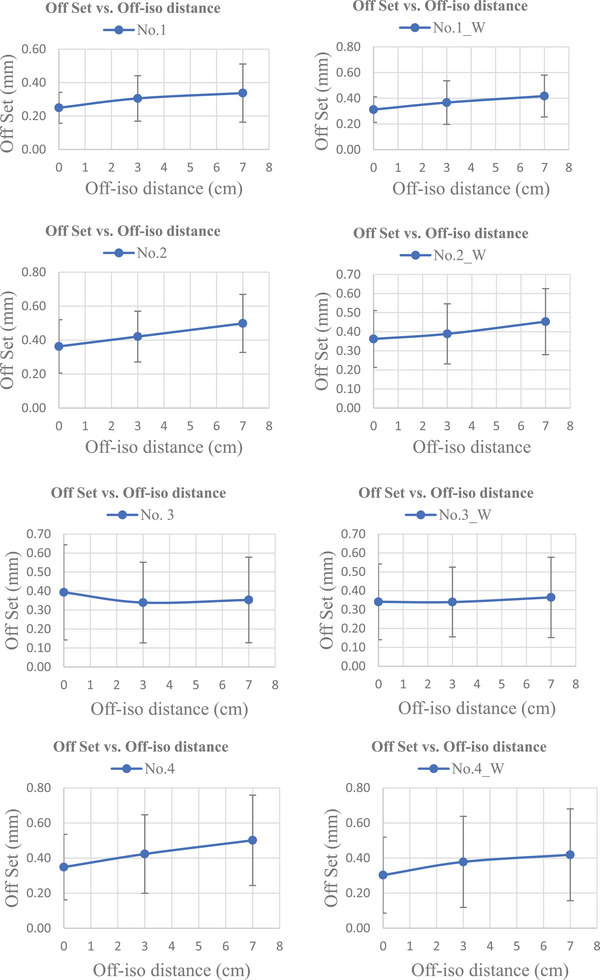

**Figure:**
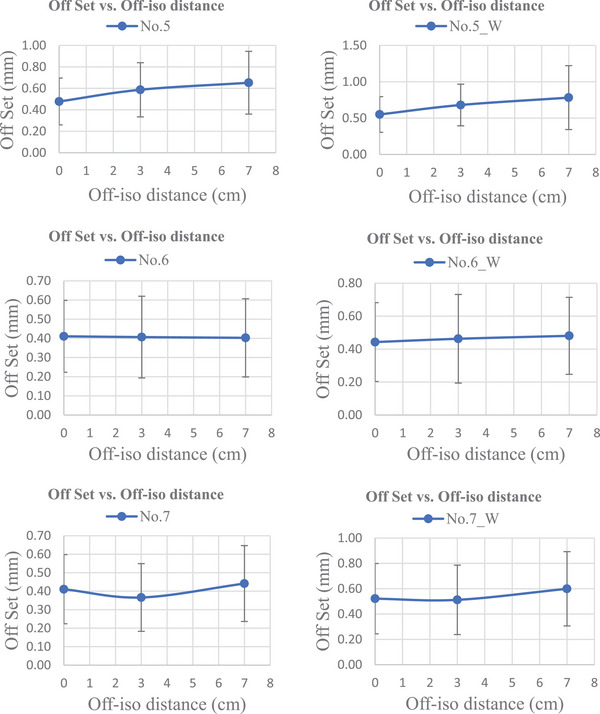

## 4 DISCUSSION

This study is the most comprehensive and accurate direct measurement of the Off‐iso Winston‐lutz test on seven linear accelerators to date of this writing. Historically, medical physicists performed the Winston‐Lutz test by aligning the test target (either a metal sphere or cubic phantom) with a Linac crosshair. However, simply aligning the test target with crosshairs is not sufficient to achieve submillimeter accuracy. The initial placement of the test target must be as close to the imaging isocenter (DICOM origin) as possible. This is because all test analysis is performed in image space. In this study, a two‐step method was developed to position all targets with high accuracy closer to their expected positions in the imaging space. In the treatment planning system, all targets exist in virtual space and are located at their designed positions with zero uncertainty. The two‐step method provides a practical strategy to relocate the targets from the planning isocenter to the imaging isocenter. This study first time quantitively investigated the accuracy of test target initial placement.

Among the seven linear accelerators included in this study, No. 1 and No. 2 were brand new TrueBeam machines with digital control systems. As shown in Figure 7, the Field‐Target Coincidence (i.e., offset) was within 1 mm at any angle set for those machines. This means that the maximum offset between target center and radiation field center was within tolerance and that all nineteen whole range cardinal GCC angle sets were suitable for delivery of SIMT‐SRS treatments when the target off‐iso distance is less than 7 cm. These angles and distance warrant the CTV to PTV 1 mm margin expansion feasible in SIMT SRS. Linacs No. 3 and No. 4 were two Varian VitalBeam machines commissioned in 2018 and 2022, respectively. Figure 7 shows that machine No. 3 was above the warning criteria at GCC angle sets (0,90,0) and (0,270,0), while Linac No. 4 exhibited similar warning signs at angle sets (0,45,45) and (0,90,0). This indicates that those warning angle sets should be avoided when choosing beam delivery arcs during the treatment planning phase. There are two measurements on each VitalBeam that were outside of the 1 mm tolerance, constituting a failure as opposed to a warning. This was likely due to measurement uncertainty. Linacs No. 5, No. 6, and No. 7 are all Varian C‐Series machines. It is worth nothing that Linac No. 6 had a major repair and re‐commissioning a few years ago due to flooding. Linacs No. 5 and No. 7 failed to meet the offset <1 mm tolerance in multiple GCC angle sets, indicating a clear failure of the off‐iso Winston‐Lutz test at those angles. This result shows that, for SIMT‐SRS planning, a 1millimeter PTV margin expansion is not adequate to ensure accurate treatment delivery. Other size margin (2 or 3 mm) must be considered and the risk of developing radionecrosis has to be assessed. The 1 mm PTV margin is still suitable for machine No. 6, which was within tolerance for all GCC angle sets except (0,0,0). The No. 7 Machine also has ExactCouch instead of the Varian IGRT couch. Intercomparison of the machines showed that ExactCouch was more suspectable to the addition of patient weight than the IGRT table. Results for each table are included in Figure 7.

Figure 8 shows the plot of average measurement results for all GCC angle sets of each Linac. The left column shows measurement results on the free table, while the right column presents measurement results with the addition of 200 pounds of weight on the table. Linacs No. 1, No. 2, No. 4, and No. 5 present a clear increase in the target offset increase as the off‐isocenter distance increased. Linacs No. 3, No. 6, and No. 7, however, showed no clear trend. The findings for Linacs No. 3, No. 6, and No. 7 are similar to those published by Wen et al.^2^ and Oliver et al.^11^ Other research has found that the trend continues until reaching an off‐iso distance of 8 cm (Yaqoub et al.) or 9 cm (Li et al.), after which the trend no longer holds. These observations necessitate more critical thinking on available measurement tools.

The off‐iso Winston‐lutz test is intended to measure the performance deficit of Linacs’ mechanical and radiation component performance. The electronic portal imaging device (EPID) is the primary method used for test. According to TG‐307,^15^ the EPID image detector has severe horns off‐axis effect: “The EPID exhibits a strong response change to the primary beam with off‐axis distance for flattened beams due to the variation in incident primary‐beam energy with off‐axis distance. This can be seen in a raw (non‐flood field corrected) EPID image which has severe horns off axi”s^15^. This off‐axis horn effect is not typically seen in clinical imaging, provided that the image detector has undergone proper dark field and flood field calibration. Per authors’ experience, most community hospitals and freestanding clinics use the PortalVision IAS3 calibration procedure to correct for off‐axis horn effect, which is often lacking in C‐Series machines. TrueBeam image calibration is much more user‐friendly and leads itself to convenient periodic calibration. To date, there is no research reporting a PortalVision calibration before the off‐iso Winston‐Lutz test. Consequently, we speculate that the trend was comprised by the horns off‐axis effect in EPID on some machines.

While this study presents the direct measurement of the off‐isocenter Winston‐Lutz test to submillimeter accuracy, it also highlights the shortcomings of current QA devices and software. On average, an experienced physicist will spend about 2 h on each test (both with and without weight), and the physicist must remodify the treatment plan for each machine. Additionally, the planned delivery time is long (about 24 min), and the cost of the necessary equipment is high. An intuitive and efficient QA system is still urgently needed to meet the demand for routine clinical off‐iso Winston‐Lutz tests, especially for community hospitals and freestanding clinics. The ideal model for a comprehensive QA system would be like the TrueBeam Cone Winston‐Lutz test module, where everything is built into the MPC package.

## 5 CONCLUSION

The direct measurement of the off‐isocenter Winston‐Lutz test was performed on seven linear accelerators using SunNuclear's MultiMet‐WL Phantom and QA Software v2.1 for image acquisition and analysis. Observations showed that the accuracy of the initial target placement directly affected the final test results. Thus, a two‐step method was developed to ensure the precise positioning of the test target to image isocenter. Among the seven Linacs, newer model machines such as the TrueBeam demonstrated greater precision than older machines and are more suited to deliver the SIMT‐SRS procedure with a standard 1 mm PTV margin expansion. Older Linacs, like the VitalBeam commissioned in 2018, were suitable for SIMT‐SRS procedures but should be routinely checked via the off‐iso Winston‐Lutz test to find optimal GCC angle sets for beam delivery. C‐Series machines are not suitable for SIMT‐SRS using a 1 mm PTV expansion. Larger margins (2 or 3 mm) should be considered instead and risk should be assessed for the development of radionecrosis.

The offset between the target center and radiation field center did not always increase with off‐iso distance for every machine. This may be attributed to the EPID's severe horn off‐axis effect. Comparing the results of the off‐isocenter WLG test with and without 200 pounds of simulated patient weight on the table, it was found that the Varian IGRT couch was less susceptible to errors introduced by patient weight than the ExactCouch. From the above results, the necessity of implementation of WLG in clinic is further enhanced in the medical physics field.

## AUTHOR CONTRIBUTIONS

Junfang Gao initiated the project, collected all data, and drafted the initial version. David Anand collected data, drew sketches, language edit, and revision format.

## CONFLICT OF INTEREST STATEMENT

The authors declare no conflicts of interest.
